# Discovering a new class of antifungal agents that selectively inhibits microbial carbonic anhydrases

**DOI:** 10.1080/14756366.2018.1516652

**Published:** 2018-10-04

**Authors:** Giannamaria Annunziato, Laura Giovati, Andrea Angeli, Marialaura Pavone, Sonia Del Prete, Marco Pieroni, Clemente Capasso, Agostino Bruno, Stefania Conti, Walter Magliani, Claudiu T. Supuran, Gabriele Costantino

**Affiliations:** a Department of Food and Drugs, University of Parma, Parma, Italy;; b Department of Medicine and Surgery, Ospedale Maggiore di Parma, University of Parma, Parma, Italy;; c Department of NEUROFARBA, Section of Pharmaceutical and Nutraceutical Sciences, University of Florence, Firenze, Italy;; d National Council of Research (CNR), Istituto di Bioscenze e Biorisorse, Napoli, Italy;; e Experimental Therapeutics Program, IFOM the FIRC Institute for Molecular Oncology Foundation, Milano, Italy

**Keywords:** Carbonic anhydrase inhibitors, antimicrobial resistance, drug design, drug discovery, antifungal agents

## Abstract

Infections caused by pathogens resistant to the available antimicrobial treatments represent nowadays a threat to global public health. Recently, it has been demonstrated that carbonic anhydrases (CAs) are essential for the growth of many pathogens and their inhibition leads to growth defects. Principal drawbacks in using CA inhibitors (CAIs) as antimicrobial agents are the side effects due to the lack of selectivity toward human CA isoforms. Herein we report a new class of CAIs, which preferentially interacts with microbial CA active sites over the human ones. The mechanism of action of these inhibitors was investigated against an important fungal pathogen, *Cryptococcus neoformans*, revealing that they are also able to inhibit CA in microbial cells growing *in vitro*. At our best knowledge, this is the first report on newly designed synthetic compounds selectively targeting β-CAs and provides a proof of concept of microbial CAs suitability as an antimicrobial drug target.

## Introduction

It is widely accepted that the use of antimicrobials allowed the control of many infectious diseases, while their misuse and overuse promoted the emergence and spreading of antimicrobial resistance (AMR)^1^^,^^2^. In a number of relevant pathogens, multidrug resistance is considered one of the most serious current threats for global health^3^^,^^4^. The infections caused by resistant microorganisms are difficult to treat, and usually require costly and sometimes toxic alternatives to common therapeutics. Problems related to AMR are expected to increase unless higher efforts are made to preserve currently available drugs and to intensify the search for new antimicrobial agents. In the drug discovery field, different strategies have been pursued in the past years to identify novel compounds able to escape existing mechanisms of resistance^5^. Cloning and high throughput screening of the genomes of many pathogens paved the way for the identification of alternative cellular pathways, as reservoir of potentially new antimicrobial targets considered essential for pathogens metabolism and replication^6–8^.

In this scenario, we embarked into multidisciplinary activities aimed at the identification and validation of new targets relevant for antimicrobial therapy^9–13^.

Recently, enzymes belonging to the carbonic anhydrase superfamily (CAs, EC 4.2.1.1)^14^ emerged as potential drug targets in antibiotic-resistant pathogens^15^^,^^16^. Such enzymes catalyse a simple, but physiologically relevant, process: the hydration of carbon dioxide to bicarbonate and protons. CAs are ubiquitously expressed in almost all living organisms and they are encoded by seven evolutionarily unrelated gene families^17^: α-CAs (present in vertebrates, bacteria, algae, and in the cytoplasm of green plants); β-CAs (in bacteria, algae, and chloroplast of monocotyledons and cotyledons); γ-CAs (mainly in Archaea and some bacteria); δ-CAs (present only in marine diatoms); ζ-CAs (in marine phytoplankton), η-CAs (identified in protozoa), and the recently discovered θ-CAs in the marine diatom *Phaeodactylum tricornutum*^18^. All of them require metal cations to convert carbon dioxide to bicarbonate. The α-, β-, δ-, η-, and perhaps θ-CAs use Zn^2+^ ions at the active site, whereas the γ-CAs are probably Fe^2+^ enzymes and ζ-class uses Cd^2+^ to perform catalysis reaction. ζ-CAs bind Cd(II) or Zn(II) within the active site and are defined as cambialistic enzymes^19^. The 3D structural folds for four out of seven CAs were determined by X-ray crystallographic studies, supporting the notion that their structural fold as well as their oligomerisation state highly differ from each other^20^.

**Figure 1.**
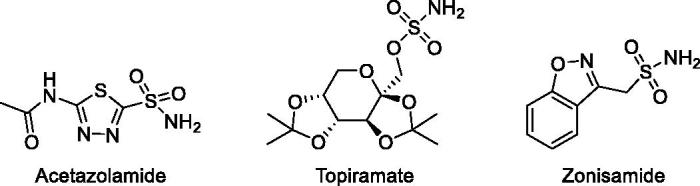
Commercially available carbonic anhydrase inhibitors.

The conversion of CO_2_ into bicarbonate and protons is implicated in different physiological and pathological processes^21^. Classical CA inhibitors (CAIs) are primary sulphonamides, represented by the general formula RSO_2_NH_2_ (Figure 1), and up to now there are 30 drugs in clinical use or in clinical development characterised by the presence of such functional groups. In addition, it has recently emerged that CAIs have a potential as anticonvulsant, anti-obesity, anticancer, anti-pain and also as anti-infective drugs^22^.

In many pathogens, CAs are essential in various steps of growth and their inhibition leads to growth impairment or growth defects^23^. Several of the already clinically approved CAIs were successfully tested *in vitro* against CA enzymes of important human pathogens^24^. However, their development as antimicrobial drugs is hampered by the side effects due to the lack of selectivity toward the human CA isoforms. In human, 15 different α-class isoforms are expressed in many tissues/organs, thus explaining the side effects, such as hepatic cirrhosis, metabolic acidosis, kidney stones deposition, and arrhythmia^25–28^, observed in using CAIs as therapeutics. Unfortunately, to date, there are no CAIs that selectively target only microbial isoforms. The disclosure of highly selective inhibitors for relevant microbial CAs would thus be of great value (i) to reduce the off-targeting of the human isoforms, dramatically reducing the occurrence of side effects; and (ii) to further confirm CA enzymes as potential antimicrobial drug targets in therapeutic interventions against drug-resistant pathogens.

**Figure 2.**
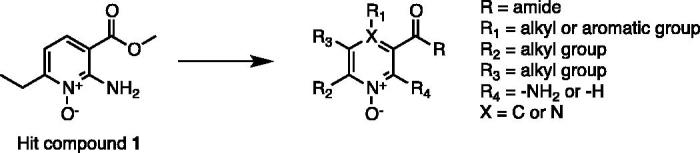
New pyridine *N*-oxide derivatives and modification sites.

In the effort to identify new chemotypes endowed with the desired pharmacological profile, we previously reported^10^ the identification of a class of CAIs characterised by the presence of a new potential Zinc Chelating Group (ZCG), which preferentially interact with microbial CA active sites over the human ones. The identified compounds showed an interesting degree of selectivity but were characterised by low µM affinity, highlighting the need to be optimised in classical medicinal chemistry cycles. We herein report the optimisation of our class of inhibitors. The previously identified hit compound (**1**, Figure 2)^10^ was used as a starting point for the hit expansion. All the newly synthesised compounds were tested against a large panel of different classes of CAs, allowing us to identify a small sub-set of compounds that inhibit β- and η-CAs with a selectivity fold greater than 80 over the human α-CAs.

The most promising compounds were investigated against *Cryptococcus neoformans* cells growing *in vitro.* Such pathogen was chosen accordingly to the following key observations: (i) this yeast is an important and worldwide distributed human opportunistic pathogen, and cryptococcosis is associated with significant morbidity and mortality, particularly in immunocompromised patients^29^^,^^30^; (ii) the spreading of resistant *C. neoformans* strains has been reported^31^, and, furthermore, the antifungal treatment for cryptococcosis is usually aggressive, toxic, and inefficient^32^; (iii) finally, it has been demonstrated that *C. neoformans* tightly depends on the activity of the β-CA Can2 for growth, CO_2_ sensing and, consequently, pathogenicity and virulence^33^^,^^34^.

The selected compounds were therefore tested against *C. neoformans* strains in different conditions (air or CO_2_-rich environment), revealing that our compounds are able to penetrate microbial cells and to engage Can2-CA thus inhibiting yeast growth.

The results highlight that it is possible to identify compounds selectively targeting different classes of CAs. The ability of our CAIs to selectively inhibit microbial growth provides a robust proof of concept that CAs can be suitable targets for drugs specifically acting on microbial pathogens, including fungi.

## Results and discussion

### Synthesised compounds

**Scheme 1.**
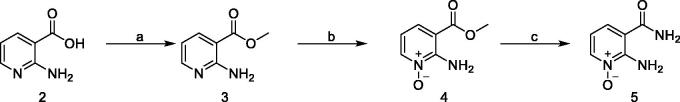
Reagents and conditions. (a) TMS-diazomethane, toluene/methanol, 0 °C, 30 min, 95%; (b) MTO, 35% aqueous H_2_O_2_, EtOH, RT, 3 h, 78%; (c) NH_4_OH, RT, 3 h, 98%.

Compounds **4** and **5** were obtained from commercially available 2-aminonicotinic acid **2**, via prior conversion into their methyl ester **3** by reaction with trimethylsilyl (TMS)-diazomethane in toluene/methanol, followed by ethyltrioxorhenium(VII) (MTO)-catalysed oxidation of the pyridine nitrogen obtaining compound **4**. The reaction of **4** with a solution of ammonium hydroxide led to desired compound **5** (Scheme 1).

**Scheme 2.**
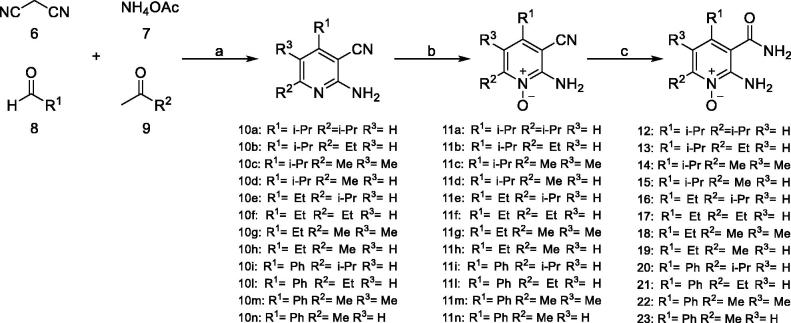
Reagents and conditions. (a) appropriate aldehyde, appropriate ketone, malononitrile, ammonium acetate, MW, 100 °C, 15 min, 37–65%; (b) MTO 35% aqueous H_2_O_2_, EtOH, RT, 3 h, 78%; (c) 10% KOH, 100 °C, 3 h, 98%.

In some cases, due to the lack of useful pyridine-based starting materials from readily available commercial sources, the *de novo* construction of the pyridine ring, with appropriate substituents, was necessary. A reported microwave-accelerated, one pot, multicomponent reaction (MCR) was exploited for the synthesis of compounds **12–23**, with slight modifications to the original procedure (Scheme 2). A fast and versatile MCR, already optimised in our laboratories, was followed^35^, which allowed to obtain, in very few steps, a series of pyridine *N*-oxide derivatives with the selected substituents at the desired positions. In this way, we also afforded an improved knowledge of the chemical space of the active site. Irradiating a tube by a microwave oven (MW) containing malononitrile (**6**), ammonium acetate (**7**), as ammonia source, an appropriate aldehyde (aromatic or aliphatic) (**8**), and an enolizable, aliphatic ketone (**9**), with neither solvent nor catalyst, for 15 min at 100 °C, provided key pyridine products **10a–i** in 37–65% isolated yields. Oxidation of pyridine nitrogen of MCR-derived intermediates (**11a–i**) followed by hydrolysis of the cyano group, afforded compounds **12–23** in good yields.

**Scheme 3.**
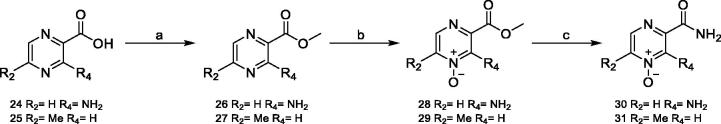
Reagents and conditions. (a) TMS-diazomethane, toluene/methanol, 0 °C, 30 min, 95%; (b) MTO, 35% aqueous H_2_O_2_, EtOH, RT, 3 h, 78%; (c) NH_4_OH, RT, 3 h, 98%.

Compounds **28**–**31** were obtained from commercially available compounds **24** and **25**, via prior conversion into their methyl esters **26** and **27** by reaction with TMS-diazomethane in toluene/methanol, and MTO-catalysed oxidation of the pyridine nitrogen to obtain desired compounds **28** and **29**. The reaction of these compounds with a solution of ammonium hydroxide led to desired compounds **30** and **31**, respectively (Scheme 3).

### *In vitro* characterisation of CAIs and structure–activity relationship (SAR) analysis

In a previous work^10^, we reported the identification of a new chemotype as prototype of new selective inhibitors for microbial CAs. The reported compounds relied on the presence of *N*-oxide pyridine ring as new potential ZCG, showing an encouraging activity, in the low µM range, and combining an interesting selectivity profile toward the human isoforms. The identified compounds represented excellent hits to be further optimised in classical medicinal chemistry optimisation cycles. Prompted by these encouraging results, we decided to start a hit-to-lead campaign aimed at improving the activity profile of our class of inhibitors, while preserving the selectivity profile toward the human CA isoforms. The main sites of scaffold modifications, reported in Figure 2, can be summarised as follow: (i) retention of the 2-amino *N*-oxide-pyridine core as main scaffold of most of synthesised compounds; (ii) modification of the ester moiety in position R with a more metabolically stable functional group; (iii) introduction of alkyl or aromatic groups of different nature in position R_1_; (iv) introduction of alkyl in position R_2_ and R_3_; (v) introduction of a nitrogen atom in position 4 (X), since the 1-*N*-oxide pyrazine ring is a scaffold described in several approved drugs (e.g. Minoxidil^36^, Acipimox^37^).

In Table 1, all the synthesised compounds are reported, which were characterised *in vitro* for their ability to inhibit different classes of CAs. The panel of CAs is composed as follows: (i) 4 α-CAs (three humans CA I, CA II, and CA IX and one bacterial from *Sulfurihydrogenibium yellowstonensis*, *Ssp*CA); (ii) 1 η-CA (from *Plasmodium falciparum, Pf*CA); and (iii) 3 β-CAs (*Vibrio cholerae*, *Vch*CA; *Malassezia globosa*, *Mg*CA; *C. neoformans*, Can2-CA). The aforementioned CAs were chosen in order to compare the activity of the synthesised compounds on human isoforms and microbial CAs representing already validated targets for antimicrobial drugs^38^^,^^39^.

**Table 1.** Synthesised compounds.


Compounds	X	R	R_1_	R_2_	R_3_	R_4_
**4**	C	–OEt	–H	–H	–H	–NH_2_
**5**	C	–NH_2_	–H	–H	–H	–NH_2_
**12**	C	–NH_2_	–*i*-Pr	–*i*-Pr	–H	–NH_2_
**13**	C	–NH_2_	–*i*-Pr	–Et	–H	–NH_2_
**14**	C	–NH_2_	–*i*-Pr	–Me	–Me	–NH_2_
**15**	C	–NH_2_	–*i*-Pr	–Me	–H	–NH_2_
**16**	C	–NH_2_	–Et	–*i*-Pr	–H	–NH_2_
**17**	C	–NH_2_	–Et	–Et	–H	–NH_2_
**18**	C	–NH_2_	–Et	–Me	–Me	–NH_2_
**19**	C	–NH_2_	–Et	–Me	–H	–NH_2_
**20**	C	–NH_2_	–Ph	–*i*-Pr	–H	–NH_2_
**21**	C	–NH_2_	–Ph	–Et	–H	–NH_2_
**22**	C	–NH_2_	–Ph	–Me	–Me	–NH_2_
**23**	C	–NH_2_	–Ph	–Me	–H	–NH_2_
**28**	N	–OEt	–	–H	–H	–NH_2_
**29**	N	–OEt	–	–Me	–H	–H
**30**	N	–NH_2_	–	–H	–H	–NH_2_
**31**	N	–NH_2_	–	–Me	–H	–H

Differently, from our previous work, *h*CA III was discharged from the analysis since our class of inhibitors proved to be completely inactive against this isoform. We introduced *h*CA IX since it is the human isoform characterised by the highest binding site similarity with *Ssp*CA (Figure S1), for which our class of compounds showed good inhibitory activity.

In Table 2, the inhibition constant (*K_i_*) values for the different CAs are reported. Accordingly to our initial hypothesis, all the investigated compounds are inactive towards the tested *h*CA isoforms. At the same time, the modifications introduced in our main scaffold led to a drop in activity against *Ssp*CA, which is a microbial α-CA. As expected and in agreement with our previous data, the activity and selectivity are conserved for *Pf*CA, which belongs to the η-class^38^ and represents an already identified target for treating malaria. Most importantly, our compounds showed a good activity profile toward three CAs belonging to the β-class^40^ from the following human pathogens *V*. c*holerae*^41^, *M*. *globose*^42^, and *C*. *neoformans*^34^.

**Table 2.** Inhibition constants (*K_i_*) of investigated compounds (Cmpd) and selectivity index toward human (CA I, CA II and CA IX) and microbial (*Ssp*, *Pf*, *Vch*, *Mg*, Can2) CA isoforms.

Compounds	Inhibition constant, Ki (μM)								Selectivity index, SI				
	α-family				η-family	β-family							
	CAI	CAII	CAIX	*Ssp*	*Pf*	*Vch*	*Mg*	Can2	*Ssp*	*Pf*	*Vch*	*Mg*	Can2
**4**	>100	>100	>100	46.9	9.2	>100	>100	>100	2.13	10.86	1	1	1
**5**	>100	>100	>100	83.6	56.6	2.1	52.8	1.7	1.68	1.76	47.62	1.89	58.82
**12**	>100	>100	>100	>100	29.0	6.8	47.9	9.5	1	3.45	14.70	2.09	10.53
**13**	>100	>100	>100	>100	9.1	2.8	>100	15.4	1	11	35.71	1	6.49
**14**	>100	>100	>100	68.0	6.0	3.2	>100	7.3	1.47	16.67	31.25	1	13.70
**15**	>100	>100	>100	59.0	66.4	8.6	>100	7.0	1.69	1.51	11.63	1	14.28
**16**	>100	>100	>100	58.5	5.5	3.4	>100	>100	1.71	18.18	29.41	1	1
**19**	>100	>100	>100	42.2	78.2	4.7	>100	17.9	2.37	1.28	21.28	1	1
**20**	>100	>100	>100	75.0	6.7	>100	46.2	>100	1.33	14.92	1	2.16	1
**21**	>100	>100	>100	>100	6.6	2.2	>100	>100	1	15.15	45.45	1	1
**22**	>100	>100	>100	64.4	9.2	34.9	>100	>100	1.55	10.87	2.86	1	1
**23**	>100	>100	>100	69.1	54.6	3.4	>100	>100	1.45	1.83	29.41	1	1
**28**	>100	>100	>100	48.3	8.1	75.9	7.1	>100	2.07	12.34	1.32	14.08	1
**29**	>100	>100	>100	>100	58.3	>100	34.9	>100	1	1.71	1	2.86	1
**30**	>100	>100	>100	>100	7.5	38.4	6.7	1.1	1	13.33	2.60	14.92	90.91
**31**	>100	>100	>100	60.8	8.6	62.0	>100	1.3	1.65	11.63	1.61	1	76.92
**AAZ**	0.25	0.012	n.a	0.005	0.17	0.451	74.0	0.01	–	–	–	–	–

**AAZ**: acetazolamide.

Such results highlight that it is possible to obtain class selectivity inside the CA super-family. Indeed, most of the modifications introduced on the main scaffold have dramatically reduced the propensity of our inhibitors to bind the α-class (the only one expressed in human) in favour to the η- and β-class.

The results reported in Table 2 reveal that for *Pf*CA (i) the introduction of the amide moiety is well tolerated; (ii) the effects of the different substituents in position R_1,_ R_2_, and R_3_ seem to be cooperative, where R_2_ and R_3_ seem to play a predominant role (**5***vs.***12–19**); (iii) the introduction of bulkier substituents in position R_1_ are in general tolerated (**20–23**). The cooperative effects observed for R_1_, R_2_, and R_3_ can be ascribed to electronic effects of the different substituents or to small changes in the ability of the different derivatives to accommodate inside the *Pf*CA active site, both affecting the ability of the *N*-oxide moiety to chelate the metal ion. Lacking of any X-ray crystal structure for *Pf*CA, it is difficult to draw firm conclusions but of great interest is the possibility to introduce bulkier substituents in position R_1_ (**20–22**), paving the way for further rounds of medicinal chemistry optimisation cycles.

**Figure 3.**
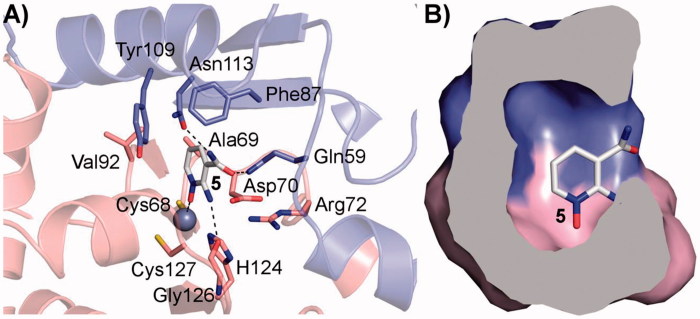
(A) Binding mode of **5** into the Can2-CA active site, depicted as pink cartoon and sticks for one Can2-CA monomer and in blue cartoon and sticks for the other monomer; (B) surface representation of the residue surrounding compound **5**, showing that small-medium substituents can be accommodated. The surface is colour-coded accordingly to (A).

On the contrary, for the β-CAs considered in this study three X-ray crystal structures (Can2-CA, PDB codes 2W3N and 2W3Q; *Vch*CA, PDB code 5CXK) are reported and can be used to rationalise the SAR profile of compounds reported in Table 2. The analysis of the X-ray crystal structures reported so far for β-CAs highlights that this sub-family can adopt two different conformations at the active site level (Figure S2)^41^: an open conformation likely prone to host small molecules and a closed conformation which is not accessible to small molecules and not catalytically active^43^. Docking studies were performed on Can2-CA (PDB code: 2W3N), since it is the only one showing an open conformation and the binding mode of compound **5** is depicted in Figure 3(A,B). The following key interactions can be observed: (i) the *N*-oxide group chelates the Zn^2+^ metal ion; (ii) the amino group in position 2 H-bonds the backbone oxygen atom of Gly126; (iii) the amide functional group in position 3 establishes two H-bond with Gln59 and Asn113, thus explaining why compounds bearing an ester in this position are inactive (**5***vs.***4**, **28** and **29**); (iv) the other side of the pyrimidine aromatic ring protrudes toward a roomier hydrophobic pocket defined by Ala69, Phe87, Val92 and Tyr109, thus explaining why only small or medium substituents are well tolerated in position R_2_ and R_3_ (**12–14**, **19***vs.***20–23, 30, 31**, Figure 3(B)); and finally (v) bulkier substituents in position R_1_ are not well tolerated in Can2CA, probably it can be due to the limited size of the roof of the pocket (Figure 3(B)). The conversion of the pyridine ring into its aza derivative pyrazine has no effects on the ability of the compounds to bind Can2-CA.

As expected, the sequence analysis comparison between *Vch*, *Mg*, and Can2-CA reveals that the enzyme active site is highly conserved inside this family. Nevertheless, important amino acidic substitutions can be identified (Figure S3), supporting the different profile activity observed for our class of inhibitors for these three β-CAs (Table 2). Relevant amino acidic substitutions are represented by: (i) Asn113 in Can2-CA is substituted with Ala and Val in *Mg* and *Vch*, respectively, thus explaining why the ester derivatives showed some degree of activity for *Mg* and *Vch* (**4**, **28**, **29**, Table 2); (ii) Ala 69 in Can2-CA is substituted with a Ser residue in *Mg,* thus explaining why hydrophobic groups in position R_2_ and R_3_ are less tolerated in *Mg* with respect to *Vch* and Can2-CA (**12–23**, Table 2); (iii) Tyr109 is substituted with a Phe in *Mg* and this modification together with the substitution of Asn113 (see point (i)) largely affects the electronic nature of the enzyme active site, thus explaining why *Mg* and *Vch* CAs less tolerate the presence of the pyrazine ring (**28–31**, Table 2); finally (iv) all the β-CAs considered are characterised by different sequence length, especially at the N-ter and active site levels, and we cannot firmly exclude that such differences can have effects on the ligand binding mechanism of the different derivatives (Figure S3).

These structural information can pave the way for further round of medicinal chemistry optimisation cycles aimed at improving the affinity of this class of inhibitors while preserving selectivity. Nevertheless, at this point the main questions which still remain to be addressed are: (i) if the inhibition of CAs by our compounds in whole cells has an effect on microbial growth and survival, and (ii) in case a phenotypic manifestation is observed, if this is linked to the inhibition of CAs.

### *Biological characterisation and target engagement of CAIs in* C. neoformans *cells growing* in vitro

The causative agent of cryptococcosis is a major opportunistic pathogen, associated with severe meningoencephalitis in immunocompromised hosts^29^. *Cryptococcus* species are inherently resistant to echinocandins^32^ and azole-resistance is emerging in *C. neoformans* strains^4^. The current gold standard therapy for cryptococcal disease presents significant side effects and is not widely available in resource-limited healthcare settings^32^^,^^44^^,^^45^. The identification of effective and specific inhibitors of *C. neoformans* CAs would thus be enormously beneficial for the development of a new class of molecules with potential anti-cryptococcal activity.

It has been previously demonstrated that the growth of *C. neoformans* can be hindered through supplementation of the potent Can2-CAIs acetazolamide (AAZ) and ethoxyzolamide (ETZ)^33^^,^^34^. However, these compounds show stronger effects on the human cytosolic isoform CA II^34^ and may lead to toxicity problems due to the off-targeting.

**Figure 4.**
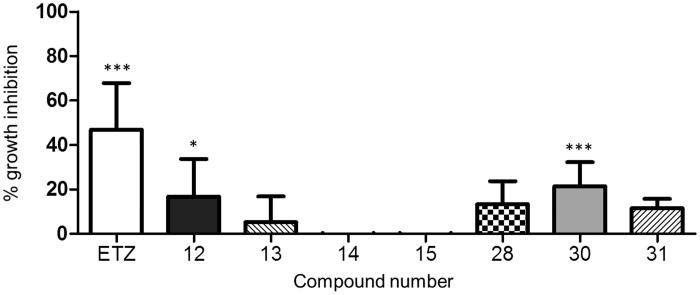
Inhibition of *C. neoformans* ATCC 6895 growth in liquid medium. Yeast cells (1.5 × 10^6^ cells/ml) were grown in YNB broth at 30 °C for 72 h, 160 rpm, in presence of the investigated compounds (3 mM) and ethoxyzolamide (ETZ), as a positive control. Growth was assessed using optical density measurements at 540 nm (OD_540_) and percent growth inhibition was calculated in comparison with cells incubated in a medium added with solvent alone (3% DMSO). The results are presented as the average of six independent experiments, each carried out in triplicate, ± standard deviation (*p < 0.05, ***p < 0.001, *vs.* control, as determined by one-way Anova with Dunnet *post hoc*).

On the other side, many CAIs that display an excellent selective activity *in vitro* against CAs from different bacterial and fungal pathogens often fail to inhibit microbial growth when challenged against the whole microorganism, probably because of the difficulty to penetrate inside the cells^46^. The effect of our inhibitors on the growth of *C. neoformans* ATCC 6895 was initially evaluated in a liquid medium in aerobic conditions. The results showed a statistically significant inhibition exerted by compound **30** (21.5%) and, to a lower extent, by compound **12** (16.9%) (Figure 4), in line with the *in vitro* inhibition of the enzyme (Table 2) and thus confirming the activity of these compounds on the whole yeast cells.

In many organisms, including *C. neoformans*, the deletion of CA encoding genes or CA inhibition leads to severe growth defects in ambient air, but CA-deficient microorganisms can grow in the atmosphere with higher CO_2_ concentration because the enzyme is dispensable in these conditions^40^. To demonstrate that the effect of our compounds was mediated by the interaction with the fungal CA, the growth of *C. neoformans* was thus assessed on agar plates supplemented with compounds **30** and **12** and incubated both in ambient air and in 5.5% CO_2_ atmosphere. In these conditions, compound **12** had no significant effect on *C. neoformans*. Interestingly, the number of colonies of *C. neoformans* grown in the presence of compound **30** in ambient air was significantly decreased by 42.9% as compared to control (Table 3).

**Table 3.** Inhibition of *C. neoformans* ATCC 6895 growth in presence of selected compounds at 37 °C in the aerobic environment or in 5.5% CO_2_.

Compound (3mM)	Growth inhibition (%) *vs.* control	
	Air	5.5% CO_2_
**30**	42.9 ± 26.4**^,^#	13.3 ± 12.4
**12**	4.2 ± 5.9	0.0 ± 0.0
**ETZ**	25.6 ± 15.6**	8.2 ± 11.2

ETZ: ethoxyzolamide.

** *p* < 0.01 *vs.* control (medium added with solvent alone).

# *p* < 0.05 *vs.* compound **30** in 5.5% CO_2_, assessed by Student’s *t* test.

**Figure 5.**
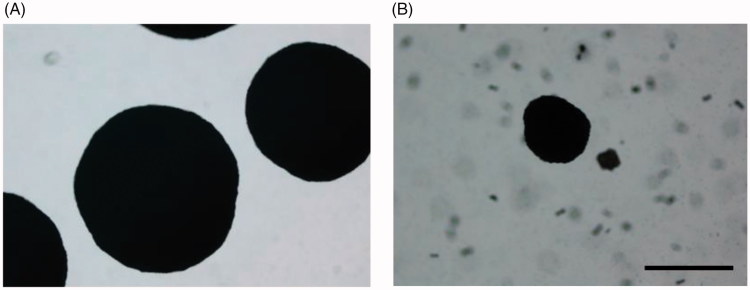
Effect of compound 30 on the size of *C. neoformans* colonies. Phase-contrast microscopy images (4×) of yeast colonies grown at 37 °C for 120 h in aerobic conditions on YNB agar plates supplemented with (A) 3% DMSO solvent alone, (B) 3 mM compound **30**. Bar: 500 µm.

The average diameter of the colonies was also significantly reduced (average diameter 263.18 ± 75.77 and 858.17 ± 99.66 µm for treated and control colonies, respectively; *P* < 0.001 as determined by Student’s *t* test; Figure 5).

**Figure 6.**
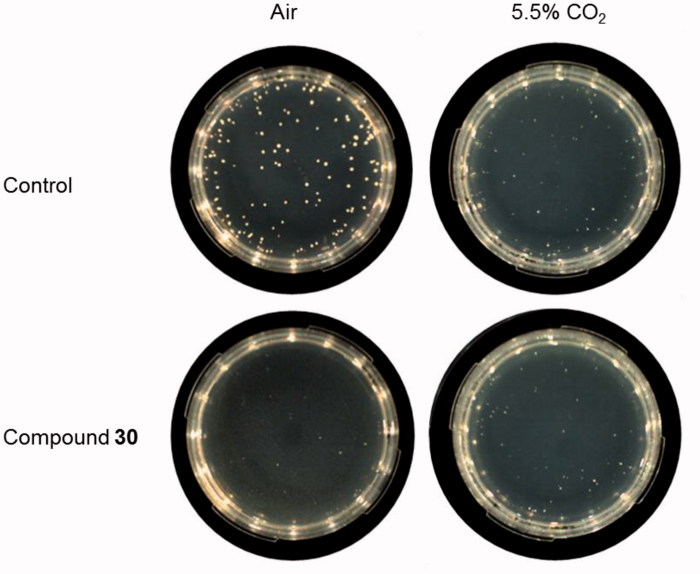
Inhibition of *C. neoformans* ATCC 6895 growth on solid medium supplemented with compound **30.** Yeast cells (200 cells/plate) were grown in YNB medium supplemented with compound **30** (3 mM), or solvent alone (control, 3% DMSO), at 37 °C for 120 h in the aerobic environment or in 5.5% CO_2_ atmosphere. Three plates for each condition were assayed in two independent experiments. Representative images are shown.

No significant growth inhibition was observed on plates added with compound **30** incubated in 5.5% CO_2_ atmosphere, proving the complementation of growth when CA activity is not essential and confirming the enzymatic target of the inhibitor. Representative images of the analysed plates are shown in Figure 6.

The concentrations used to inhibit the growth of *C. neoformans* were higher than those needed for the inhibition of the purified CA enzyme, probably because of the barrier role of the fungal cell wall. Although the pharmacokinetic properties of the compounds should be optimised for therapeutic purposes *in vivo*, these results represent, at our knowledge, the first demonstration of the activity of selective β-CAs inhibitors against microbial whole cells.

## Conclusions

The results of this study highlight the possibility to design and synthesise compounds that selectively target different classes of CA. Indeed, the newly reported inhibitors preferentially interact with the β- and η-CAs over the α ones. The most promising compound, in terms of affinity and selectivity, when tested against *C. neoformans*, an important human pathogen, revealed its ability to inhibit microbial growth through CA inhibition, providing a robust proof of concept that CAs are targets that may be specifically engaged by molecules lacking host toxicity. Our compounds may thus represent the prototype of selective CAIs which may be used for the expansion of the current arsenal of anti-infective drugs.

## Materials and methods

### Chemistry section

All the reagents were purchased from Sigma-Aldrich (Milan, Italy), Alfa Aesar (Riga, Latvia), and Enamine at reagent purity and, unless otherwise noted, used without any further purification. Dry solvents used in the reactions were obtained by distillation of technical grade materials over appropriate dehydrating agents. MCRs were performed using CEM Microwave Synthesizer-Discover model. Reactions were monitored by thin layer chromatography on silica gel-coated aluminium foils (silica gel on Al foils, SUPELCO Analytical, Sigma-Aldrich) at 254 and 365 nm. Where indicated, intermediates and final products were purified by silica gel flash chromatography (silica gel, 0.040−0.063 mm), using appropriate solvent mixtures. ^1^H NMR and 13C NMR spectra were recorded on a BRUKER AVANCE spectrometer at 400 and 100 MHz, respectively, with TMS as an internal standard. ^1^H NMR spectra are reported in this order: multiplicity and number of protons. Standard abbreviations indicating the multiplicity were used as follows: s = singlet, d = doublet, dd = doublet of doublets, t = triplet, q = quadruplet, m = multiplet, and br = broad signal. HPLC/MS experiments were performed with an Agilent 1100 series HPLC apparatus, equipped with a Waters Symmetry C18, 3.5 μm, 4.6 mm ×75 mm column and an MS: Applied Biosystem/MDS SCIEX instrument, with API 150EX ion source. HRMS experiments were performed with an LTQ ORBITRAP XL THERMO apparatus.

All compounds were tested at 95% purity or higher (by HPLC/MS).

Synthetic procedures and compounds characterisation are reported in Supporting Information.

### Molecular modelling

The X-ray crystal structure of Can2-CA was used to pursue docking studies by means of Glide^47^. The protein was prepared with the protein preparation tool of Maestro^47^, and the docking grid was centred on the Zn^2+^ metal ions. Compound **5** was prepared with the atom builder tool of Maestro^47^ each docking run was carried out using the standard precision (SP) method and the van der Waals scaling factor of nonpolar atoms was set to 0.8, as previously reported^10^. The binding mode having the highest score and able to recapitulate the reported SAR was selected.

### *In vitro* CA inhibition assay

An Sx.18Mv-R Applied Photophysics (Oxford, UK) stopped-flow instrument has been used to assay the catalytic activity of various CA isozymes for CO_2_ hydration reaction^48^. Phenol red (at a concentration of 0.2 mM) was used as indicator, working at the absorbance maximum of 557 nm, with 10 mM Hepes (pH 7.5, for α- and η-CAs) or TRIS (pH 8.3, for β- and γ-CAs) as buffers, 0.1 M Na_2_SO_4_ (for maintaining constant ionic strength), following the CA-catalysed CO_2_ hydration reaction for a period of 10 s at 25 °C. The CO_2_ concentrations ranged from 1.7 to 17 mM for the determination of the kinetic parameters and inhibition constants. For each inhibitor at least six traces of the initial 5–10% of the reaction have been used for determining the initial velocity. The uncatalysed rates were determined in the same manner and subtracted from the total observed rates. Stock solutions of inhibitors (10 mM) were prepared in distilled–deionised water and dilutions up to 1 nM were done thereafter with the assay buffer. Enzyme and inhibitor solutions were pre-incubated together for 15 min (standard assay at RT) prior to assay, in order to allow for the formation of the enzyme–inhibitor complex. The inhibition constants were obtained by non-linear least-squares methods using PRISM 3 and the Cheng–Prusoff equation, as reported earlier^10^^,^^49^. All CAs were recombinant proteins produced as reported earlier by our groups^50–53^.

### Cryptococcus neoformans inhibition assays

The growth inhibition of *C. neoformans* ATCC 6895 in presence of compounds **12**–**15**, **28**, **30**, and **31**, and ETZ as a positive control, was evaluated in YNB medium (2% glucose, 1 × Difco yeast nitrogen base without amino acids), pH 7.0. Yeasts were pre-grown at 37 °C on Sabouraud dextrose agar (SDA) plates for 48 h. The compounds were resuspended in hot DMSO (100 °C) according to their solubility (concentrations ranging from 300 to 100 mM). Growth assays in liquid medium were performed on 96-wells microtiter plates. Yeast cells (1.5 × 10^6^ cells/mL) were incubated in 200 μL of YNB with 3 mM compounds in ambient air for 72 h at 30 °C, 160 rpm. Three wells for each condition were prepared together with controls (medium alone and medium with DMSO). Growth was measured spectrophotometrically, recording the optical density at 540 nm (OD_540_) before (*t_0_*) and after incubation. After baseline correction, percent growth inhibition was calculated using the following formula: 100 – [(OD_540_ at 72 h – OD_540_ at *t_0_*)/(OD_540_ in 3% DMSO at 72 h – OD_540_ in 3% DMSO at *t_0_*) × 100]. Six independent experiments were performed. For growth assay in a solid medium, 200 cells of *C. neoformans* were spread on YNB agar supplemented with selected compounds at a concentration of 3 mM and incubated at 37 °C in air and 5.5% CO_2_ atmosphere. After 120 h the colony forming units (CFUs) were counted. Percent growth inhibition was calculated in comparison to controls as previously described^54^. Three plates for each condition were assayed in two independent experiments. The average colony diameter per plate was determined from microscope images of 20 randomly selected colonies (NIS-Elements D 3.00, SP6 Imaging software). Statistical analyses were performed by GraphPad Prism 5 software. A *p* values <0.05 was considered significant.
